# The Mechanisms of Ferroptosis Under Hypoxia

**DOI:** 10.1007/s10571-023-01388-8

**Published:** 2023-07-17

**Authors:** Xin Gao, Wei Hu, Dianlun Qian, Xiangfeng Bai, Huilin He, Lin Li, Shibo Sun

**Affiliations:** 1 Department of Pulmonary and Critical Care Medicine, First Affiliated Hospital, Kunming Medical University, No. 295, Xichang Road, Wuhua District, Kunming, 650032 China; 2 2020 Clinical Medicine Class 6, Kunming Medical University, Kunming, 650500 China; 3 Department of Cardiology, The First Affiliated Hospital of Kunming Medical University, Kunming Medical University, Kunming, 650032 China; 4 Department of Cardiothoracic Surgery, First Affiliated Hospital, Kunming Medical University, Kunming, 650032 China

**Keywords:** Ferroptosis, Hypoxia, Hypoxia-inducible factor, Lipid peroxidation, System Xc

## Abstract

Ferroptosis is a new form of programmed cell death, which is characterized by the iron-dependent accumulation of lipid peroxidation and increase of ROS, resulting in oxidative stress and cell death. Iron, lipid, and multiple signaling pathways precisely control the occurrence and implementation of ferroptosis. The pathways mainly include Nrf2/HO-1 signaling pathway, p62/Keap1/Nrf2 signaling pathway. Activating p62/Keap1/Nrf2 signaling pathway inhibits ferroptosis. Nrf2/HO-1 signaling pathway promotes ferroptosis. Furthermore, some factors also participate in the occurrence of ferroptosis under hypoxia, such as HIF-1, NCOA4, DMT1. Meanwhile, ferroptosis is related with hypoxia-related diseases, such as MIRI, cancers, and AKI. Accordingly, ferroptosis appears to be a therapeutic target for hypoxia-related diseases.

## Introduction

Ferroptosis is a newly discovered form of iron-dependent programmed cell death that is induced by the accumulation of iron-mediated lipid peroxidation (Fuhrmann and Brune 2022). It is reported that small molecule erastin induces an iron-dependent cell death pattern from apoptosis, necrosis and autophagy in tumor cells with oncogene RAS mutation, which is officially named as ferroptosis (Dixon et al. 2012). Ferroptosis is mainly characterized by intracellular iron accumulation and lipid peroxidation, and is mainly related to intracellular iron accumulation, glutathione (GSH) depletion, glutathione peroxidase 4 (GPX4) inactivation, and increasing lipid peroxidation (Dixon et al. 2012; Yang et al. 2016). Increasing studies confirm that ferroptosis is related to multiple signaling pathways and participates in the regulation of many diseases. Meanwhile, accumulating evidence suggests that ferroptosis plays an important role in hypoxia-related diseases such as myocardial infarction (MI), acute kidney injury (AKI), neurodegenerative disorders, ischemia stroke (IS), hypoxic tumors (Zhao et al. 2021b; Zhou et al. 2022). In the injuries of many organs such as the heart, brain, and kidney, ferroptosis is reported to play an essential role in inducing the occurrence of diseases by influencing iron metabolism or lipid peroxidation. In neurodegeneration disease, ferroptosis is inhibited by reducing the iron content to suppress the production of lipid peroxides (Masaldan et al. 2019). In cancer, ferroptosis is induced to suppress the development of non-small cell lung cancer (NSCLC) cells (Bebber et al. 2021). Many drugs for cancer treatment have been found to induce ferroptosis to control the development of cancer cells, suggesting that the induction of ferroptosis reaches the intention of treating cancer (Lachaier et al. 2014; Zhao et al. 2021a).

Hypoxia is caused by internal and external conditions, such as high altitude, ischemia–reperfusion injury, and solid tumors (Ow et al. 2018; McClelland and Scott 2019; Chen et al. 2022). Many reasons may cause hypoxia in people. For example, there is insufficient blood flow to partial areas in tumors, or decreasing in hemoglobin in tissues. Hypoxia regulates iron-related proteins to effect iron concentration and lipid peroxidation. Hypoxia-inducible factor-1 (HIF-1) is regulated by hypoxia to increase iron uptake, thereby affecting ferroptosis sensitivity. Hypoxia also regulates ferroptosis by Nrf2/HO-1 signaling pathway, p62/Keap1/Nrf2 signaling pathway. Besides, epigenetic modification plays a role in regulating ferroptosis under hypoxia, such as miRNA, lncRNA, methylation. There is an important relationship between ferroptosis and hypoxic diseases. Hypoxia induces cardiomyocyte ferroptosis through mitochondrial dysfunction caused by calcium overload, resulting in impaired cardiac function. Hypoxia also regulates ferroptosis in brain by inducing abnormal iron metabolism and oxidative stress. In addition, ferroptosis is related with cancers, organ damage caused by ischemia, and coronavirus disease-19 (COVID-19). In this review, we aim to describe the regulatory mechanisms of ferroptosis and the relationship between ferroptosis and hypoxia-related diseases.

## Regulation of Ferroptosis

### Iron Metabolism

Iron absorbed in body is mainly Fe^3+^ which loads onto the transferrin (TF) in serum and then combine with transferrin receptor (TfR) in the cell membrane. Subsequently, the TF-Fe/TfR complex is endocytosed into the cell. With the help of six-transmembrane epithelial antigen of prostate 3 (STEAP3) in the endosome, Fe^3+^ is reduced to Fe^2+^ and then Fe^2+^ is released into cytoplasm by divalent metal transporter 1 (DMT1) (Hu et al. 2022).It is confirmed that the ferritin in cytoplasm is the main iron storage protein. Ferritin is composed of ferritin light chain (FTL) and ferritin heavy chain 1 (FTH1). FTH1 mainly oxidizes Fe^2+^ into Fe^3+^ and the FTL mainly makes next Fe^2+^ enter the ferroxidase site (Fuhrmann et al. 2020). The deficiency of iron leads to nuclear receptor coactivator 4 (NCOA4) recognizing the ferritin and then the ferritin moves to lysosomes (Li et al. 2020b). Subsequently, the iron ions in ferritin are released into cytoplasm. Abnormal iron metabolism causes iron overload, which leads to the Fenton reaction (Hu et al. 2022). Iron chelating agents and nitrogen oxides are inhibitors of Fenton reaction. For example, deferoxamine (DFO) is combined with Fe^3+^ to form iron amine complexes to reduce the impact of iron overload, ultimately reducing unstable iron in cells and then inhibiting the Fenton reaction in the process of ferroptosis (Ben Ismail et al. 1994; Cheng et al. 2021). In addition, TEMPO inhibits the formation of hydroxyl radical to block the Fenton reaction, which may inhibit the process of ferroptosis (Shi et al. 2017). The Fenton reaction increases generation of reactive oxygen species (ROS) which ultimately causes ferroptosis (Zhou et al. 2018; Xu et al. 2021c). The concentration of TF in serum of patients with hypoxic diseases was significantly higher than that of healthy people (Koistinen et al. 2000; Li et al. 2022c). Hypoxia causes the increase of erythropoietin (EPO), which in turn leads to the increase of serum TF, eventually leading to abnormal iron metabolism to promote ferroptosis. Besides, hypoxia enhances HIF-1 levels to promote the concentration of transferrin to regulate ferroptosis (Li et al. 2022c). Consequently, iron metabolism plays an important role in ferroptosis.

### Regulation Pathways of GPX4

GPX4 is a selenoprotein antioxidant enzyme also called phospholipid-hydroperoxide glutathione peroxidase (PHGPx) (Hu et al. 2022; Wei et al. 2022). GPX4 oxidizes GSH into glutathione (GSSG) and reduces toxic L-OOH to non-toxic L-OH, which controls the spread of lipid peroxide and maintains the stability of cytomembrane (Xu et al. 2021c). In addition, the knockdown of GPX4 increases intracellular ferrous iron and ROS, and finally causes ferroptosis (Wei et al. 2022). Simultaneously, excessive iron decreases the expression of GPX4 and solute carrier family 7 member 11 (SLC7A11), which accelerates the occurrence of ferroptosis. GSH is composed of cysteine, glutamic acid and glycine, and is a co-factor with GPX4 in the catalysis of peroxides. The decrease of cysteine causes depletion of GSH, which leads to inactivation of GPX4 and then ferroptosis occurs (Yang et al. 2016). Studies present that inhibition of GPX4 induces ferroptosis (Hou et al. 2021; Liu et al. 2022). It is suggested that the GPX4 inhibitor triggers ferroptosis in cancers (Fan et al. 2021a; Li et al. 2021c; Zhang et al. 2022b). In addition, RAS-selective lethal 3 (RSL3), an inhibitor of GPX4, bonds with GPX4, which effectively leads to lipid peroxides and downregulates ion GPX4 in glioblastoma, resulting in ferroptosis of glioblastoma (Li et al. 2021c; Hu et al. 2022). Bufotalin (BT), a novel GPX4 inhibitor, increases the degradation of GPX4 and intracellular Fe^2+^ level, causing ferroptosis in non-small cell lung cancer cells (Zhang et al. 2022b). Correspondingly, the up-expression of GPX4 inhibits ferroptosis. It is presented that the platycodin D (PD) treatment in diabetic nephropathy (DN) and curculigoside (CUR) treatment in ulcerative colitis (UC) is mainly through suppressing ferroptosis with the elevation of GPX4 (Wang et al. 2020a; Huang et al. 2022a). GPX4 expression is activated by activating transcription factor 4 (ATF4) and androgen receptor (AR). Heat shock protein family A member 5 (HSPA5) increased by activation of ATF4 inhibits lipid peroxidation in ferroptosis by protecting against GPX4 degradation in cancer cells (Zhu et al. 2017). AR activation increases GPX4 and decreases ROS production to affect hypoxia-mediated ferroptosis in MIR (Zhang et al. 2022c). Accordingly, GPX4 is a regulator of ferroptosis.

### System Xc-

System Xc- is a part of the heterodimeric amino acid transporter (HAT) family and is a cystine/glutamate antiporter system (Chen et al. 2021c; Liu et al. 2021a). System Xc- in the cell membrane is composed of two subunits, the solute carrier family 7 member 11 (SLC7A11) and the SLC3A2 (Liu et al. 2021a). Cystine is converted into cysteine which is one of the components of GSH. The GSH is reduced with the dysfunction of system Xc-, which leads to the decrease of GPX4 and the accumulation of lipid peroxidation, then the ROS increases, and ultimately ferroptosis is induced (Chen et al. 2021c; Li et al. 2022a). It is reported that IFN-γ inhibits System Xc- through down-regulating SLC3A2 and SLC7A11 to induce ferroptosis (Kong et al. 2021). Naringenin (NAR) regulates system Xc- to inhibit ferroptosis in Myocardial ischemia–reperfusion injury (MIRI) rats (Xu et al. 2021b). ATF induced by oxidative stress inhibits the expression of SLC7A11 to repress the system Xc-, resulting in ferroptosis (Wang et al. 2020b; Feng et al. 2021a). In colorectal cancer cells, 2-imino-6-methoxy-2H-chromene-3-carbothioamide (IMCA) downregulates the expression of SLC7A11 and decreases cysteine to result in ferroptosis (Zhang et al. 2020). Besides, prevotella histicola (P. histicola) activates the anti-ferroptosis system Xc-/GPX4 axis to reduce ferroptosis in ethanol-induced gastric mucosal lesions (EGML) (Wang et al. 2023). Canagliflozin (Cana), an anti-diabetes drug, also promotes the system Xc-/GPX4 axis to inhibit ferroptosis, attenuating cardiovascular diseases (Du et al. 2022). At present, system Xc- inducers still need more studies to clarify the relationship between system Xc- and ferroptosis. Accordingly, system Xc- may be a potential regulator of ferroptosis.

### Nuclear Factor Erythroid 2-Related Factor 2 (Nrf2)

Nrf2 is a key transcription factor in antioxidation and plays an important role in the regulation of intracellular iron concentration (Hu et al. 2022). Nrf2 binds to Kelch-like-ECH-associated protein 1 (Keap1) in the cytoplasm, and then Nrf2 is inactivated by ubiquitination (Xu et al. 2021c; Hu et al. 2022). However, under oxidative stress, Nrf2 is released from Keap1 and translocates into the nucleus, subsequently interacts with the antioxidant response element (ARE) to drive the expression of antioxidant genes and then cells are protected from oxidative stress (Xu et al. 2021c). It is reported that Nrf2 inhibits ferroptosis through enhancing the cellular antioxidant ability (Wang et al. 2020c). The activation of Nrf2 reduces ROS, increases FTH1 and GPX4 mRNA expression to balance oxidative stress and then ferroptosis is inhibited (Qiu et al. 2020; Zhang et al. 2021). Besides, Nrf2 plays an indirect role in ferroptosis through regulating lncRNA and miRNA. Decreased expression of noncoding myocardial infarction-related transcripts (lncRNA) promoted ferroptosis by regulating Nrf2 (Wang et al. 2022). LncRNA metastasis-associated lung adenocarcinoma transcript 1 (MALAT1) prevents Nrf2 from moving into the nucleus by increasing Keap1 levels, ultimately leading to ferroptosis (Radhakrishnan and Kowluru 2021). Inhibiting microribonucleic acid 27a (miRNA-27a) in ischaemic stroke brain issue may inhibit ferroptosis by upregulating Nrf2 (Zhang et al. 2022a). Bach1, repressing some of Nrf2 target genes, inhibits MUFA biosynthesis to induce ferroptosis (Xie et al. 2023).

Moreover, p62/Keap1/Nrf2 signaling pathway is a key pathway to inhibit ferroptosis. P62 is an adaptor protein and is also a cytoplasmic protein induced by oxidative stress. P62 to activate Nrf2 by combining with Keap1, leading to the upregulation of GPX4 and GSH, thereby protecting cells from oxidative stress. Besides, p62 activation increases the degradation of Nrf2 and scavenges lipid peroxides to inhibit ferroptosis (Li et al. 2020a). The accumulation of p62 which is caused by inhibition of autophagy, leads to Keap1 dysfunction, and then prevents Keap1-mediated degradation of Nrf2, activating the Nrf2 pathway to protect cells from ferroptosis (Ji et al. 2015). Thus, Nrf2 plays an important role in regulating ferroptosis.

### Heme Oxygenase-1 (HO-1)

HO-1, an anti-inflammatory and antioxidant intracellular enzyme, metabolizes heme to ferrous iron, carbon monoxide (CO) and biliverdin (Wang et al. 2020c; Xu et al. 2021c). Over-expression of HO-1 leads to Fenton reaction, GSH depletion, and lipid peroxidation in clear cell renal carcinoma (ccRCC), which causes ferroptosis in ccRCC (Han et al. 2022). Ferroptosis inhibition effectively reduces sepsis-induced acute kidney injury (SAKI), which is related to Nrf2/HO-1 signaling pathway activated by melatonin (MEL) (Qiu et al. 2022). Elevating the expression and activity of HO-1 increase the levels of free iron and expression of subsequent ferritin (Xu et al. 2021a; Machado et al. 2022). Chronic HO-1 overexpression causes excessive iron in normal cells, promoting intracellular toxicity and cell death (Nitti et al. 2018). However, adequate HO-1, caused by Nrf2, protects cells from toxicity.

The Nrf2/HO-1 signaling pathway is an important signaling pathway to protect cells against oxidative stress. HO-1 is regulated by Nrf2. When Nrf2 is activated, it promotes the expression of HO-1. The upregulation of HO-1 expression regulates antioxidant enzymes which transforms free radicals into water and molecular oxygen, reduces oxidative stress damage and the production of oxidation products, exerting antioxidant effects (Loboda et al. 2016). In recent years, increasing studies demonstrate the Nrf2/HO-1 signaling pathway inhibits ferroptosis. Activating Nrf2/HO-1 signaling pathway reduces hypoxia/reoxygenation (H/R)-reduced ferroptosis of cardiomyocytes by icariin (ICA), while levistilide A (LA) activities Nrf2/HO-1 signaling pathway to promote ferroptosis in breast cancer (BC) (Liu et al. 2021b; Jing et al. 2022). Meanwhile, Nrf2/HO-1 signaling pathway is activated by hypoxia and Nrf2 overexpression in hypoxia-induced HTR-8/SVneo cells activates Nrf2/HO-1 signaling pathway and decreases oxidative stress and ferroptosis (Wang et al. 2021c).

### Hypoxia-Inducible Factor (HIF)

HIF is a key transcription factor mediating adaptation to hypoxia, hypoxia-inducible factors (HIFs) consist of HIF-1, HIF-2 and HIF-3 (Urrutia and Aragones 2018; Li et al. 2022d). HIF-1 and HIF-2 regulate ferroptosis in a context-dependent manner. However, whether HIF-3 is associated with ferroptosis is unclear. HIF-1 may suppress ferroptosis in AKI by prompting mitophagy, decreasing mitochondrial redox homeostasis, limiting mitochondrial respiration and limiting mitochondrial damage (Li et al. 2022d). Besides, HIF-1 regulates the gene levels of SLC7A11, and knocking down HIF-1 decreases SLC7A11 protein in rat brain to lead to ferroptosis (Hsieh et al. 2017). HIF-1 is decreased in hepatic stellate cell (HSC) by sorafenib, and then reduces SLC7A11 expression, then leads to GPX4, GSH depletion and increases ROS level in HSC, and ultimately causes HSC ferroptosis (Yuan et al. 2022). HIF-1 is inhibited to promote ferroptosis through expression of the core circadian clock gene period 1 (PER1) (Yang et al. 2022b). What’s more, activation of HIF-2 may upregulate iron regulatory and increase ROS to cause ferroptosis in colorectal cancer (CRC) (Singhal et al. 2021). Consequently, HIF play an important role in the regulation of ferroptosis.

### Endoplasmic Reticulum Stress (ER)

Recently, studies have shown that endoplasmic reticulum stress plays an important role in ferroptosis. The endoplasmic reticulum mainly maintains cell homeostasis and participates in protein synthesis (Iurlaro and Munoz-Pinedo 2016). Abnormal protein processing under stress conditions causes an unfolded protein response (UPR), and then leads to endoplasmic reticulum stress (ER) (Iurlaro and Munoz-Pinedo 2016). ER may promote ferroptosis. PERK, a marker protein in ER period, is inhibited, prominently restraining ferroptosis induced by dextran sulfate sodium (DSS) (Dixon et al. 2014). In addition, ferroptosis induced by erastin is accompanied by the occurrence of ER response (Park et al. 2019). Therefore, the combined use of ferroptosis inducer and endoplasmic reticulum stress inhibitor is of great significance for the treatment of cancer. In summary, endoplasmic reticulum is closely related to ferroptosis, and the mechanism needs further study.

**Fig. 1:**
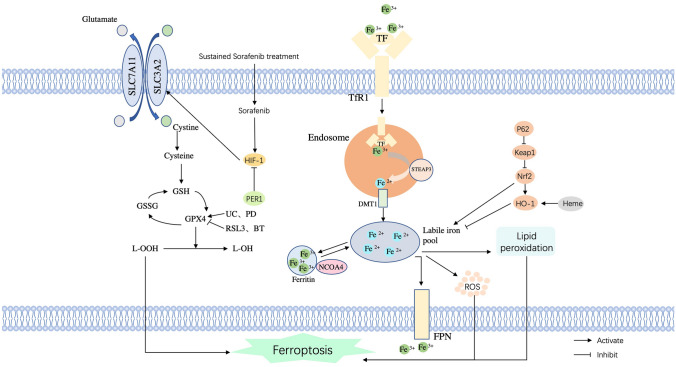
The regulatory pathways of ferroptosis

The mechanical pathways of ferroptosis are shown in Fig. 1.

## Regulations Associated with Ferroptosis Under Hypoxia

Studies suggest that hypoxia affects iron concentration, regulation of iron-related proteins, regulation of HIF-1 and other factors, leading to the accumulation of ROS and the occurrence of ferroptosis (Christova and Templeton 2007; Feng et al. 2021b). Multiple pathways are activated during this process, such as Nrf2/HO-1 signaling pathway (Liu et al. 2021b), p53/TfR1 pathway (Tang et al. 2021), Nrf2-Mediated Stress-Defense Pathway (Tao et al. 2022), Nrf2/HIF-1/TF signaling pathway (Li et al. 2020c), Egr-1/miR-15a-5p/GPX4/ferroptosis signaling pathway (Fan et al. 2021b). Among these pathways, ROS, GPX4 and GSH are mainly affected, which cause ferroptosis.

**Fig. 2:**
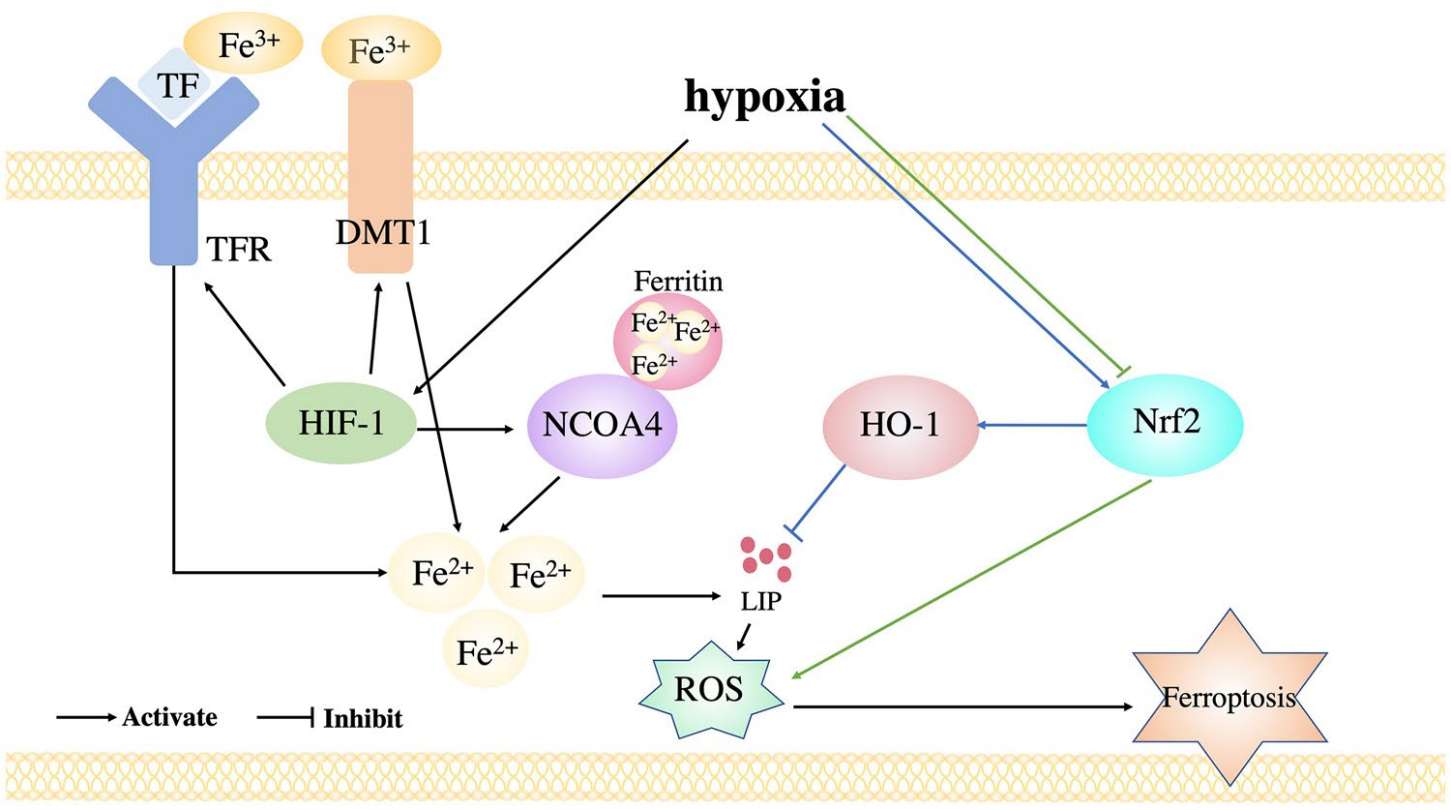
Hypoxia regulates HIF-1 and Nrf2 to regulate ferroptosis

Hypoxia mainly regulates HIF to regulate ferroptosis. Hypoxia enhances HIF-1 which up-regulated TfR and DMT1 to increase iron uptake, which effects the sensitivity of cells to ferroptosis (Xiong et al. 2022). Meanwhile, HIF-1 increases the transcription of SLC7A11 and HO-1, which both protect from ferroptosis through decreasing ROS and increasing GSH (Feng et al. 2021b; Lin et al. 2022). Moreover, it is reported that HIF-2 increases the expression of perilipin 2 (PLIN2) and hypoxia-inducible lipid droplet-associated protein (HILPDA) to increase lipid accumulation, oxidative stress, and then enhance ferroptosis (Singhal et al. 2021). The mechanism between HIF induced by hypoxia and ferroptosis remains largely unknown and needs further investigation. Furthermore, hypoxia inducing Nrf2 plays an important effect in ferroptosis (Potteti et al. 2021). Hypoxia downregulates Nrf2 in mouse tubular epithelial cells (MTEC) to cause ferroptosis (Huang et al. 2022b). The down-regulation of Nrf2 induced by hypoxia leads to the unbalanced of intracellular oxidation and antioxidant system, and then results in the increase of intracellular ROS and finally causes ferroptosis (Wang et al. 2021c). Besides, hypoxia also increases the activity of Nrf2 which increases the expression of HO-1 to protect from ferroptosis (Liu et al. 2021b; Wang et al. 2021c) (Fig. 2).

Additionally, hypoxia regulates ferritin, stearoyl-CoA desaturase 1 (SCD1), ELAV-like protein 1 (ELAVL1) and carbonic anhydrase 9 (CA9) to effect ferroptosis. Ferritin, composed with FTL and FTH1, stores intracellular Fe2^+^. Hypoxia induces FTL increasing, NCOA4 decreasing, FTH1 increasing (Fuhrmann et al. 2020; Liu et al. 2020a; Chen et al. 2023). They are the main regulators of ferritin. Hypoxia decreases the expression of NCOA4 which mediate the degradation of ferritins, and then increases iron storage to protect cells from ferroptosis (Fuhrmann et al. 2020; Ni et al. 2021). Increasing expression of FTH under hypoxia protects cells from ferroptosis (Fuhrmann et al. 2020). Hypoxia increases FTL under hypoxia, mainly storing Fe2^+^, reducing intracellular Fe2^+^ and inhibiting ferroptosis (Consoli et al. 2022; Chen et al. 2023). Moreover, hypoxia increases the expression of SCD1 which decreases ferroptosis through ferroxidase activity and generates monounsaturated fatty acid (MUFA) to protect from ferroptosis (Gao et al. 2021; Luis et al. 2021). MUFA inhibits ferroptosis by down-regulating transferrin receptor to reduce intracellular iron content (Qi et al. 2022). Additionally, hypoxia induces the expression of ELAVL1 which activates the ferritinophagy and then promotes ferroptosis (Chen et al. 2021b). ELAVL1 binds to SLC7A11 mRNA and maintains its stability. High expression of SLC7A11 inhibits ferroptosis by transporting cysteine (Lin et al. 2022).

Furthermore, it is reported that hypoxia increases CA9 which blocks ferritin-mediated iron storage and increases lipid peroxidation by inhibiting to reduce oxidative stress and then ferroptosis (Li et al. 2019b). In addition, epigenetic modification plays an important role in regulating ferroptosis under hypoxia. Gene methylation is involved in the regulation of hypoxia on ferroptosis. CBSmRNA -destabilizing lncRNA (lncRNA-CBSLR) induced by hypoxia integrated with YTH domain family protein 2 (YTHDF2) to decrease CBSmRNA stability, reducing the methylation of the Acyl-CoA synthetase long-chain family member 4 (ACSL4) and leading to degradation of ASCL4, which is conducive to protect gastric cancer (GC) cells from ferroptosis (Yang et al. 2022a). In the hypoxia environment caused by I/R, USP7 inhibition promotes DNM-1 mediated methylation of FMR1 to inhibit ferroptosis, attenuating I/R-induced renal injury(Dong et al. 2022).In addition, miRNA is involved in the regulation of ferroptosis by hypoxia. Hypoxia induces miR-214-3p upregulated to enhance ferroptosis through inhibiting malic enzyme 2 (ME2) in neonatal rat cardiomyocytes (NRCMs) (Liu et al. 2023). LncRNA also is involved in the regulation of ferroptosis caused by hypoxia. Hypoxia induces lncRNA-PMAN overexpression, and then leads to the stability of SCL7A11 improved, eventually inhibiting ferroptosis (Lin et al. 2022). At present, the relationship between epigenetic modification and ferroptosis needs more reaches to be further explored.

## Ferroptosis and Hypoxic Diseases

### Ferroptosis and Myocardial Ischemia–Reperfusion Injury (MIRI)

MIRI refers to the cardiac function damage resulted from recovery of blood supply in a short time after partial or total myocardial coronary artery occlusion, which often occurs in acute myocardial infarction, coronary heart disease (Abudunaibi et al. 2015; Dong et al. 2019; Li et al. 2021b).

Hypoxia caused by ischemia induces calcium overload which may lead to mitochondrial dysfunction and thus induce cardiomyocyte ferroptosis (Jiang et al. 2016). Cytosolic calcium overload increases mitochondrial uptake of calcium ions, and then causes the mitochondrial permeability transition pore (MPTP) opened, which impaired ATP synthesis, mitochondrial swelling, and ROS increased (Kwong 2017). Calcium overload-induced mitochondrial dysfunction leads to ROS increased to induce ferroptosis. Increased ROS leads to ferroptosis through generating Fenton reaction decreasing GPX4 and Nrf2, and decreasing HIF-1 caused by hypoxia. Meanwhile, hypoxia damages the mitochondrial electron transport chain (ETC), and then increases the ROS to induce ferroptosis (Zhao et al. 2023). Moreover, HIF overactivation caused by hypoxia upregulates TfR expression and then causes iron overload during MIRI, which ultimately induces ferroptosis (Zhang et al. 2019). Accordingly, calcium overload, excessive of ROS, and iron overload induced by HIF result in the occurrence of ferroptosis. Consequently, ferroptosis is closely related to cardiomyocyte damage under hypoxia in MIRI.

### Ferroptosis and Ischemic Stroke (IS)

IS is mainly caused by focal cerebral ischemia and hypoxia is induced by cerebral blood flow obstruction (Xu et al. 2022). Hypoxia induced by IS increases the expression of ferritin and TfR1, and then leads to increase of iron uptake by neurons, which resulting in increase of iron in cells, and then ferroptosis occurs (Lan et al. 2020). Meanwhile, the absence of ceruloplasmin under hypoxia condition may effect iron metabolism and increase oxidative damage, which is conductive to cause iron accumulation-induced ferroptosis (Ryan et al. 2018).

It is reported that increased glutamate under hypoxia leads to ferroptosis through disrupting intracellular iron homeostasis to injury the brain (Li et al. 2017). In addition, accumulation of glutamate inhibits cystine by inhibiting system Xc-, and then decreases GSH, subsequently promoting ferroptosis through ATF4-mediated ferroptotic genes in IS (Speer et al. 2013). Oxidative stress induced by hypoxia causes excessive ROS accumulation to induce Nrf2 activation, then increases GSH, SCL7A11 and GPX4 to protect cells from ferroptosis in IS (Kwak et al. 2002; Shibata and Kobayashi 2008; Dodson et al. 2019). Meanwhile, increased glutamate under hypoxia also increases ROS to active Nrf2, which inhibits ferroptosis (Shibata and Kobayashi 2008).

**Fig. 3:**
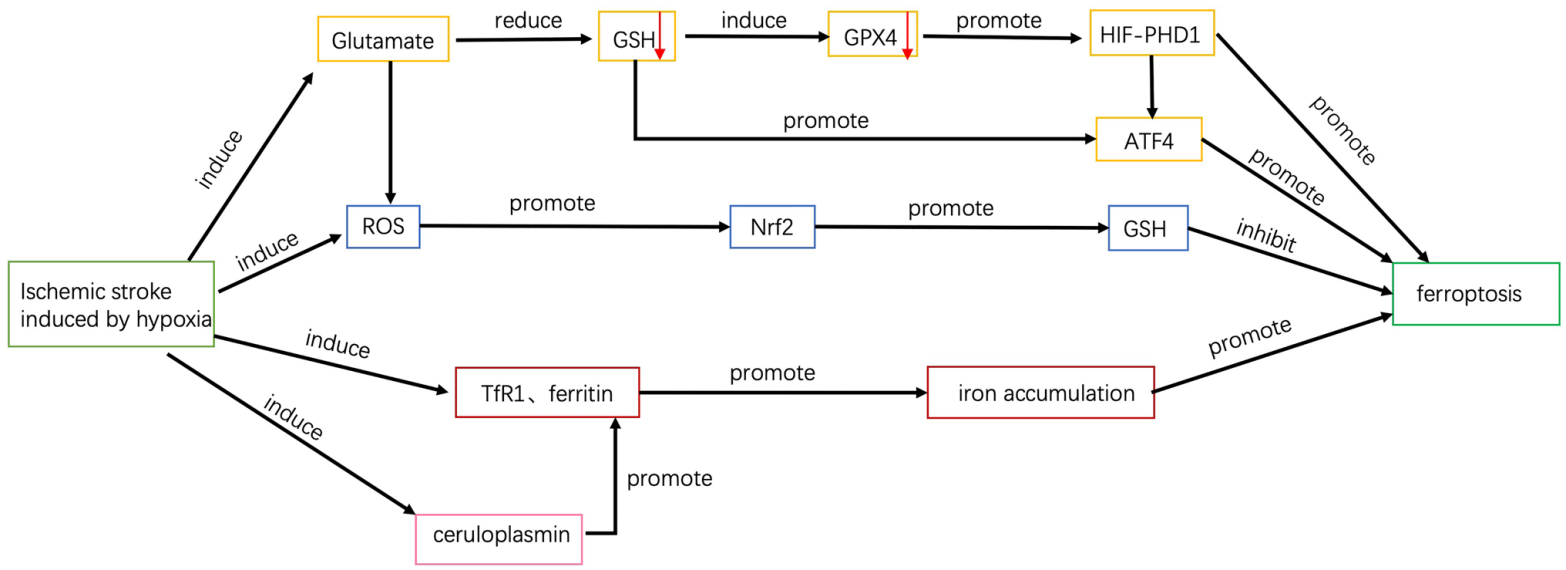
Regulations associated with ferroptosis in ischemic stroke

The regulations associated with ferroptosis in IS are shown in Fig. 3.

### Ferroptosis and Neurodegenerative Disorders

Neurodegenerative disorders are mainly present that the progressive loss of selectively vulnerable populations of neurons leads to abnormal cognitive behaviors of patients, which is common in Alzheimer’s disease (AD), Parkinsonian disorders (PD), Amyotrophic lateral sclerosis (ALS), and so on. Studies suggest that hypoxia causes the reduction of ATP synthesis and the generation of ROS damage cells, and then leads to the dysfunction of mitochondrial and the disorder of oxidative phosphorylation, resulting in ferroptosis in neurodegenerative disorders (Marques et al. 2017). AD is a common neurodegenerative disorder that is related with ferroptosis (Bao et al. 2021). Reduction of cerebral blood flow causes hypoxia in AD and the hypoxia may activate the Toll-like receptor 4 (TLR4) pathway, and then decrease the levels of SLC7A11 and GPX4, thereby leading to ferroptosis (Kimura et al. 1991; Lang et al. 2019).

### Ferroptosis and Acute Kidney Injury (AKI)

AKI is a clinical syndrome caused by rapid decline of renal function in a short time due to various reasons (Li et al. 2021a). Hypoxia caused by blood flow interruption is the main cause of AKI, which is related to ferroptosis (Longo et al. 2017). Hypoxia may lead to the up-regulation of ELAVL1which is interact with cold-induced RNA binding protein (CIRBP), and then activate ferritinophagy to result in ferroptosis (Sui et al. 2021). In addition, hypoxia down-regulates Nrf2 and upregulates SLC7A11 to result in occurrence of ferroptosis (Huang et al. 2022b). Meanwhile, hypoxia increases ROS and prevents autophagy of GPX4 to induce ferroptosis in AKI (Chen et al. 2021a). Accordingly, the ferroptosis may be involved in AKI through hypoxia. However, more researches are needed to explore the relationship between ferroptosis and hypoxia in AKI in the future.

### Ferroptosis and Cancers

Cancer mortality is high, but the mechanism is unclear. Accumulating evidence shows that the occurrence of cancer is related to ferroptosis, ferroptosis is inhibited in hepato-cellular carcinoma, pancreatic cancer, gastric cancer and other cancers. SLC7A11 is upregulated by BRCA1-associated protein 1 (BAP1) inactivation in cancer cells, which increases the uptake of cystine and the synthesis of GSH to develop the growth of cancer by inhibiting ferroptosis (Zhang et al. 2018). SLC7A11 is overexpressed to inhibit ferroptosis in lung cancer cells and pancreatic ductal adenocarcinomas (PDACs) (Badgley et al. 2020; Wang et al. 2021a). P53 promotes ferroptosis by repressing SCL7A11 to inhibit cancer development (Liu et al. 2020b). Besides, the relationship between cancer and ferroptosis is connected with hypoxia.

Hypoxia causes hypoxia regions on account of poor blood flow in many solid tumors (Takahashi 2011; Li et al. 2021d). It is reported that hypoxia is related to the relationship between SLC7A11 and ferroptosis. Hypoxia increases ELAVL1 by HIF-1 upregulation, and ELAVL1 then combines with SLC7A11 to improve the expression of SLC7A11, thereby promoting cancer cells growth by inhibiting ferroptosis (Lin et al. 2022). Meanwhile, hypoxia increases SLC7A11 by inhibiting methyltransferase-like 14 (METTL14) to suppress ferroptosis by decreasing ROS, which promotes hepatocellular carcinoma (HCC) progression (Fan et al. 2021c). Additionally, hypoxia stimulates the activity of HIF-1 transcription which increases TfR1 and DMT1 to resist ferroptosis in cervical cancer (CC) cells (Xiong et al. 2022). However, iron regulator protein 1 (IRP1) may inhibit hypoxia-induced DMT1 and control intracellular iron levels to regulate ferroptosis in HepG2 cells (Christova and Templeton 2007). The mechanism between ferroptosis and cancer under hypoxia is still unclear, which needs to be explored.

Cancer stem cells (CSCs) have strong self-renewal, diffusion and metastasis, and resistance to various forms of anticancer therapy, which can easily lead to tumor recurrence (Katoh 2017). It is reported that the expression of transferrin and TFR1 in CSCs are higher than non-CSCs, which illustrates that CSCs uptake more iron than non-CSCs from microenvironment (Schonberg et al. 2015). Thus, iron transporting in CSCs is enhancing. More importantly, the increasing of iron uptake caused by CD44 overexpression and the decreasing iron efflux caused by downregulating FPN in CSCs cause intracellular iron to be higher than non-CSCs. Ferroptosis in CSCs may be an excellent target therapy for cancer (Cosialls et al. 2021).

Hypoxia may promote cancer growth by inhibiting ferroptosis, which causes difficulties in cancer treatment. The effect of radiation on ferroptosis is mainly reflected in radiotherapy. Radiotherapy is one of the important treatments for malignant tumors, and the induction of ferroptosis is also one of the important factors. Radiotherapy suppresses SLC7A11 via activating ATM and increases lipid oxidative to cause the ferroptosis of tumor cells (Lang et al. 2019). Owing to cancer under hypoxia, the effect of radiotherapy treatment is not significant (Su et al. 2022). However, ferroptosis may play an important role in cancer treatment. Sorafenib inhibits SCL7A11 to suppress cancer by inducing ferroptosis (Li et al. 2022b). Hence, ferroptosis may be an effective targeted therapy for hypoxic cancer.

### Ferroptosis and Other Hypoxia-Related Diseases

Coronavirus disease-19 (COVID-19) is a new type of explosive disease characterized by pneumonia and acute respiratory distress syndrome (ARDS) which is accompanied with hypoxia and leads to multiple organ failure (Beckman et al. 2020; Jacobs et al. 2020). Hypoxia may antagonize the iron transporter which causes abnormal iron metabolism, and then leads to ferroptosis (Naidu et al. 2023). It is reported that hypoxia induced by COVID-19 may increase the ferritin in circulation and cause iron deficiency which cause the occurrence of oxidative stress and lipid peroxidation, resulting in ferroptosis (Lechuga et al. 2021; Ondic et al. 2021; Suresh et al. 2021; Wang et al. 2021b). Pneumonia is the primary caused by COVID-19. Pneumonia induced by COVID-19 causes atelectasis resulting in hypoxia (Rahman et al. 2021).

Intestinal I/R injury is another hypoxia-related disease. It is reported that hypoxia induced by Intestinal I/R injury decrease GPX4 activity and GSH levels, and then leads to ferroptosis (Deng et al. 2021). Moreover, hypoxia caused by intestinal I/R injury induces the expression of ACSL4 and the ACSL4 contributes to ferroptosis (Tarhan et al. 2011; Li et al. 2019a). However, the mechanism between ferroptosis and hypoxia caused by intestinal I/R injury needs further exploration.

## Conclusions

Ferroptosis is caused by iron-dependent accumulation of lipid peroxidation and the increase of ROS. The related pathways between ferroptosis under hypoxia mainly include Nrf2/HO-1 signaling pathway, p62/Keap1/Nrf2 signaling pathway. The Nrf2/HO-1 signaling pathway is currently a research hotspot in hypoxia-related diseases. However, the relationship between the p62/Keap1/Nrf2 signaling pathway and ferroptosis in hypoxia-related diseases still needs further research, and is a potential direction for future research. However, more researches on the signal pathways of ferroptosis under hypoxia are needed. Meanwhile, some factors also participate in the occurrence of ferroptosis under hypoxia, such as HIF-1, NCOA4, DMT1. Hypoxia-induced activation of HIF-1 has been shown to be closely related to ferroptosis. Because HIF-1 plays a role in ferroptosis in a context-dependent manner under hypoxia, the connection between HIF-1 and ferroptosis under hypoxia needs to be sorted out. Meanwhile, ferroptosis is related with hypoxia-related diseases, such as MIRI, cancers, and AKI. The research progress in ferroptosis and MIRI, IS, AKI, and cancers is rich, but research progress in ferroptosis and intestinal I/R injury still needs further study. In addition, hypoxic environment may inhibit the occurrence of ferroptosis and promote cancers growth, resulting in the unexpected effect of radiotherapy. It is needed that find a way to use ferroptosis to maximize the effect of radiation therapy. Inducing ferroptosis in ferroptosis-prone CSCs by controlling iron accumulation may be an excellent targeted therapy. Accordingly, ferroptosis appears to be a therapeutic target for hypoxia-related diseases.

## Data Availability

Not applicable.
